# Widening perspectives on regenerative processes through growth

**DOI:** 10.1038/npjregenmed.2016.15

**Published:** 2016-12-08

**Authors:** Stephanie H Nowotarski, Alejandro Sánchez Alvarado

**Affiliations:** 1 Howard Hughes Medical Institute, Chevy Chase, MD, USA; 2 Stowers Institute for Medical Research, Kansas City, MO, USA

## Abstract

Defining the basic mechanisms behind regeneration requires comparison to both development and homeostasis. How is organ size achieved in animals during normal development, and how is it reconstituted in animals capable of regenerating organs and body parts lost to injury? Are the mechanisms regulating size and allometry evolutionarily conserved? In recent years, discoveries in the fields of signalling, physiology, developmental biology and regeneration using a growing and diverse collection of model organisms have begun to shed mechanistic insight into these problems. Growth, central to embryonic development, tissue homeostasis and regeneration, was the unifying concept at the recent Molecular and Cellular Basis for Growth and Regeneration Keystone meeting.

## Introduction

**Figure 1:**
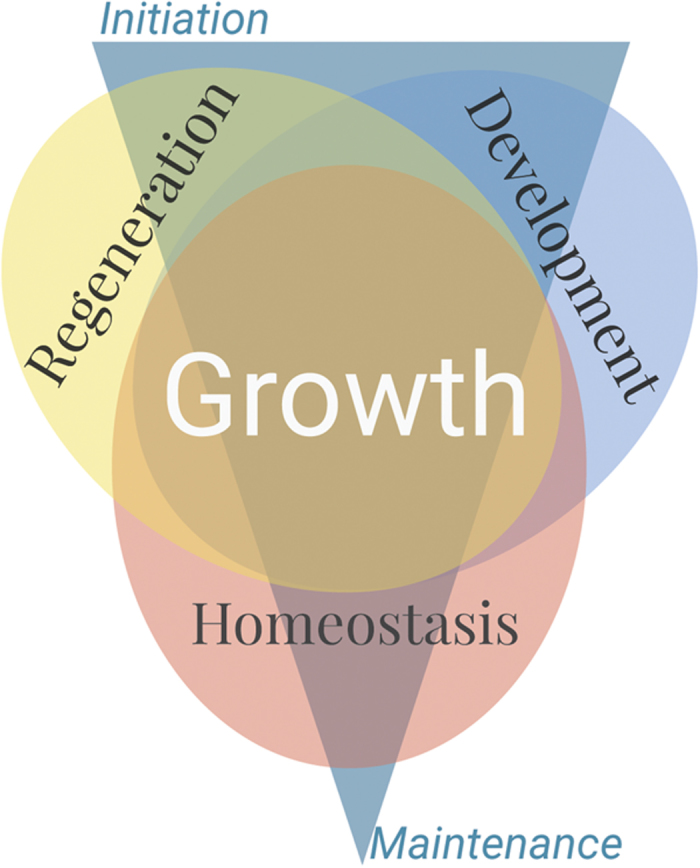
Central to regeneration, development, and homeostasis, growth was an excellent inclusive element in this meeting. Although regeneration and development are often characterised by growth or large growth potential, homeostasis is characterised by tight growth regulation and scalar/size maintenance.

The complex, awe-inspiring process of regeneration has captured scientific imagination since Aristotle. The process by which an animal senses wounding, initiates a non-scarring response, and then harnesses developmental programs to regrow a functional, properly proportioned organ or limb is an astounding biological feat accomplished by an adult organism. Fast forward two millennia to the Molecular and Cellular Basis for Growth and Regeneration Keystone Meeting at Breckenridge, CO (10–14 January), the second Keystone meeting focusing on regeneration, and you find a room full of scientists applying modern technology to these age-old questions. The first regeneration meeting, in 2011, notably united work from many regenerative organisms.^1^ This sophomore meeting, organised by Valentina Greco, Duojia Pan and Alejandro Sánchez Alvarado, retained the organismal diversity of the first and significantly broadened the regenerative perspective by comparing and contrasting it to development and homeostasis, specifically through the lens of growth. As a feature shared by all three states (regeneration, development, and homeostasis), the inclusion of growth proved highly successful, creating an inclusive, unifying and ultimately stronger meeting through its breadth and depth (Figure 1). While continuing to strengthen the inherent link between regeneration and development, this meeting also highlighted how many aspects of growth control, including mechanical forces, pattern formation, variability, and regulation of stem cells, significantly influence these processes.

## Definitions: comparing and contrasting regeneration to development and homeostasis

By directly comparing development and regeneration, we can begin to understand each process respective of the other. Although in the recent past we may have accepted a distinct definition of development, it is becoming increasingly clear the lines between embryonic development, regeneration, wound healing, homeostasis and even tumorigenesis are becoming significantly blurred. These interwoven relationships and framework for investigation was clearly represented at this meeting. One example came from Tatjana Piotrowski, who discussed work comparing recently published roles of Notch and Wnt in zebrafish lateral line neuromast regeneration^2^ to a developing story about neuromast deposition in embryonic development. The promotion of proliferation by Wnts in conjunction with the suppressive role of Notch in regeneration is notably different in early zebrafish development. Alejandro Sánchez Alvarado also used juxtaposition of development, homeostasis and regeneration to shed light on two stories, one unpublished on ontogeny of stem cells in planarian embryos (*Schmidtea mediterranea*) and another on mechanisms of their maintenance and repopulation under challenge in adult worms.^3^ Plants were represented by Kenneth Birnbaum, who discussed single cell RNAseq methods to understand patterning in regenerating *Arabidopsis* root tips, finding regeneration largely recapitulated embryonic developmental stages.^4^ In a final, beautiful example, Joachim Wittbrodt unified development, growth, and regeneration through his description of medaka eye morphogenesis using live imaging. He not only demonstrated how eye morphogenesis behaves much like gastrulation, but also explored how adult stem cells remodel during scalar lifetime growth through elegant modelling of physical constrains.^5^

Live imaging was also used to capture regenerative responses and initiation of growth in other organisms and contexts. Ken Poss described a newly developed technique of time-lapse clonal imaging with overlaid reconstruction of multiple clones, suggesting considerable heterogeneity in adult stem cells in the regenerating zebrafish tail fin.^6^ Matt Gibson presented the highly regenerative sea anemone *Nematostella vectensis* as a key model for understanding epithelial morphogenesis and growth regulation through an evolutionary lens using live imaging. Other talks that utilised sophisticated imaging techniques include complementary talks given by Aaron Mertz from Elaine Fuch’s lab and Valentina Greco. Aaron Mertz described newly published work detailing how live imaging with a spinning disc microscope revealed spatial organisation of the developing mouse epidermis.^7^ Valentina Greco used two photon confocal imaging to reveal striking spatiotemporal kinetics of cell migration and proliferation of intact and wounded epidermis in live adult mice.^8^ Elena Ezhkova shed light on the epigenetics regulating murine skin cell differentiation.^9^

Thinking about regenerative growth with respect to developmental growth begs another comparison: regenerative growth versus homeostatic growth control/maintenance. Maintaining or achieving homeostatic growth control is important in the context of wounding, regeneration and tumorigenesis. This universality underlines the need for a plastic response by adult somatic cells to sense their environment and respond appropriately. Like Valentina, Joseph Rodgers used response to injury in the adult mouse as a paradigm to investigate the reentry of quiescent cells into the cell cycle.^10^ How adult cells in a tissue maintain their number was addressed by Jayaraj Rajagopal, who used insult to basal lung cells in the mouse to reveal adult stem cells provide a niche for their progeny, ensuring tissue homeostasis and regeneration.^11^ Both Rajagopal and David Moore took this idea of plasticity and addressed growth with respect to tumorigenesis.

## Regulation: from the nucleus of stem cells to the maternal-zygotic transition

Individual cellular behaviors underpin growth and regenerative tissue responses and, in turn, are driven by regulation of transcription and translation. Probing the mechanism of cell fate specification through transcriptional regulation, Charles Sagerström discussed recent work describing how poised Tale factors control transcription of the *hoxb1a* locus in zebrafish.^12^ Understanding how histone modifications work to establish different transcriptional states in stem cells was addressed by Elisabeth Duncan from the Sánchez Alvarado lab in describing her work on how different H3K4 histone methyltransferases have different genomic targets in planarian neoblasts.^13^ Taking a macroscopic view of the nucleus, Julie Baker focused on the relationship between nuclear architecture and regeneration and presented visually striking unpublished work describing chromatin remodelling in early *Xenopus* wound response and regeneration. Finally, Antonio Giraldez presented compelling work from Ariel Bazzini looking at the role of codon usage in regulation of the maternal to zygotic transition in zebrafish.^14^ This work unveiled a novel regulation of gene expression operating through codon optimality, a concept that will likely be of great interest to many.

## Scaling: the role forces play in regeneration and growth

In thinking about mechanisms governing growth and scaling in developmental contexts, morphogen signalling gradients often come to mind first. Eric Hill, in Chris Petersen’s laboratory, addressed how Wnt and Notum contribute to brain size scaling in planaria.^15^ Jochen Rink also discussed work aiming to understand how long-range gradient patterning relates to scaling in planaria.

Alternatively, but not mutually exclusive to morphogen gradients, the idea of forces mediated through adhesion and cytoskeletal elements to inform and enforce growth was also showcased using several tissues in *Drosophila*. At the level of single cell division, Marco Gonzales Gaitan explained work on microtubules and diffusion-based mechanisms of asymmetric division in sensory precursor cells.^16^ Norbert Perrimon described unpublished work focusing on mechanical regulation of homeostasis in the gut epithelium via osmoregulation and mechanotransduction. Also on the whole tissue level, Yohanns Bellaïche described patterning of cell division across the dorsal thorax using a combination of large scale live imaging and modelling, revealing the Hertwig rule (cells divide parallel to longest axis) is enforced through tricellular junctions.^17^

Expanding creatively on both large-scale modelling and the idea of forces playing integral roles in growth and patterning, L. Mahadevan described an elegant modelling approach to the relationship between form and function at the meso-scale level of organs. Work out of his laboratory focuses on spatiotemporal growth and pattern formation. Both mathematical models and diverse physical models were used to reveal the physical basis for patterning in a variety of tissues from pollen tubes to the mouse brain.^18–20^

## Differences: evolutionary approaches to understanding shape

Taking a broader perspective, several engaging talks applied molecular mechanisms to evolutionary questions of growth, pattern, shape and function. Hopi Hoekstra commenced the meeting with an engaging keynote describing unpublished work on the mechanisms of patterning among and across rodent species.^21^ The idea of trans-species variation also drove Richard Schneider’s talk, which demonstrated through elegant neural crest cell transplants that jaw size is cell autonomous across avian species.^22^

Conversely, variation within species was addressed by Arkat Abzhanov, whose lab impressively uses a wide range of approaches from fieldwork to modelling to elucidate mechanisms of regulation in beak growth and shape in Darwin’s finches.^23^ Elaine Ostrander also described intra-specie variation on perhaps the best documented and most relatable of examples: the many breeds of domestic dog. Her lab harnesses the benefits of large-scale comparative genomics with well-documented AKC pedigrees to uncover the molecular basis for breed differences like fur, jaw and leg length and body size.^24^

What can we learn by comparing adaptations within a species versus differences that define species? David Kingsley incorporated both intra- and inter-species variation into a beautiful two-part story about important roles the loss of regulatory DNA has in evolutionary changes in vertebrates. The first story described selective pressure driving drastic phenotypic changes to marine sticklebacks through loss of regulatory regions when populations are isolated in fresh water.^25^ The second addressed a deeply fundamental question: what makes humans human? This work used comparative genomics to identify human-specific loss of regulatory DNA and then mouse genetics to demonstrate its functional consequences on the neocortex.^26^

## Immunity: innate connections to growth and regeneration

The breadth and timeliness of this meeting captured several emerging themes. One variable that likely affects development, homeostasis, wound healing and regeneration is the immune response. A field in its own right, how the immune response affects and is integrated into growth and regeneration emerged as a high interest area. Bruce Edgar discussed the roles of Egf, Ras and MapK signalling pathways in *Drosophila* gut regeneration after infection with bacteria.^27^ In larval development, Andreas Bergman talked about work linking reactive oxygen species and immune cells known as hemocytes to apoptosis-induced proliferation in the eye imaginal disc.^28^ Duojia Pan discussed recent work revealing the hippo pathway functions in the innate immune response through Yorki promoting *IkB* transcription in a Toll-mediated bacterial response.^29^ In a closely related talk, Laura Johnston presented work on Toll and cell competition in the *Drosophila* wing.^30^

The link between immunity, regeneration and growth was not limited to *Drosophila*, as Nadia Rosenthal discussed the balancing act of inflammatory scarring versus cell replacement and regeneration in both axolotl limbs and the mammalian heart, in which macrophages have an integral role.^31^ Shishir Biswas, from Ashley Seifert’s group, also discussed the interesting tradeoff between scarring and epimorphic regeneration in describing his current work comparing expression of the matrisome between the scarring *Mus musculus* and the regenerating *Acomys cahirinus* (African spiny mouse).

## Endoreplication: sizing up growth

Another emerging theme was the role of endoreplication in tissue growth, repair and regeneration. Despite knowledge of polyploidy for over a century, our understanding of its physiological function in diverse organisms still remains to be understood. It is clear that endoreplication is coupled to large cell size and differentiated cells, suggesting polyploidy serves some specialized function.^32^ This meeting captured several instances in which endoreplication had functional roles. Bruce Edgar discussed regulation of enterocyte endoreplication and work out of Wallace Marshall’s lab from Pranidhi Sood described a single celled, macro-nucleated organism capable of regeneration, *Stentor coeruleus*. However, two talks really explored the relationship between ploidy and spatial problem-solving by differentiated cells. Mary Baylies discussed size and scaling of multinucleate *Drosophila* larval muscles, which assemble through fusion events and the positioning of their endoreplicated nuclei.^33^ Her presentation of polyploid nuclear spatial territories during developmental growth was a delightful juxtaposition to what happens in response to injury of post mitotic adult tissue; Vicki Losick presented elegant work in the adult *Drosophila* epidermis describing how ventral abdominal wounds are sealed using a combination of cell fusion/ syncytium formation and polyploidy.^34^

## Conclusion

Regeneration and growth both pose complex problems that require consideration with respect to development, homeostasis and disease to begin to fully understand how each work. The Keystone meeting on Molecular and Cellular Basis for Growth and Regeneration effectively leveraged the integration of a host of models and diverse perspectives from different fields, biological scale, and techniques to address common questions and provide a platform for integral discussion of both advances and difficulties. As we’ve thankfully long passed the point of integration and overlap of scientific approach disciplines (i.e., developmental biologists incorporating and relying on molecular and biochemistry approaches), the current challenges facing the fields of regeneration and growth (and others) are what host of tools are out there and what methods on the sliding scale of molecular to evolutionary biology can and should one apply to their own problem. It is not a surprise that some of the best new ideas come from passing comments and wildly different ideas and perspectives. The breadth of this meeting provided a stage ripe for these types of moments and showcased topics like immunity and endoreplication as areas we will need to keep in mind when approaching growth and regeneration. It will be grand to see this meeting grow further and continue to generate ideas and collaborations.
